# Complications of Open Tibial Fracture Management: Risk Factors and Treatment

**DOI:** 10.5704/MOJ.1703.006

**Published:** 2017-03

**Authors:** JYC Lua, VH Tan, H Sivasubramanian, EBK Kwek

**Affiliations:** Department of Orthopaedics, Tan Tock Seng Hospital, Singapore

**Keywords:** open tibial fractures, infection, complications, antimicrobial

## Abstract

Open tibial fractures result in high rates of complications. This study aims to elucidate the risk factors causing these complications, and suggest antimicrobial regimens based on the organisms grown in post-operative infections. Over a period of five years, 173 patients had sustained open tibial fractures and undergone operative treatment at a single institution. All surgical data was gathered retrospectively through online medical records. Thirty-one patients (17.9%) had sustained post-operative bony complications, while infective complications were reported in 37 patients (21.4%). Patients with Gustilo type III fractures were found to be more than three times as likely to sustain post-operative infective (p=0.007) or bony (p=0.015) complications, compared to Gustilo type I or II fractures. The fracture location and time taken to fixation did not significantly affect the complication rate, but results were trending towards significance. The commonest cause of infective complications were hospital-acquired organisms, such as Methicillin-resistant staphylococcus aureus (40.5%). Closer monitoring of patients sustaining high grade Gustilo open fractures, as well as antimicrobial prophylaxis for both hospital-acquired organisms and environmental contaminants, will result in the best outcome for patients. Further studies with larger sample sizes are warranted, to determine the significance of fracture location and time taken to fixation on complication rates.

## Introduction

Tibial fractures are the most common long bone fractures, with around 25% being open fractures ^1^. The majority of open tibial fractures result from high velocity trauma such as road traffic accidents and falls from height. The management of these fractures can be complex due to the relative lack of soft tissue coverage and blood supply of the tibial shaft^2^. Prognosis depends on the amount of initial bone displacement, comminution, and soft tissue injury. Advanced bone reconstruction and soft tissue coverage is usually required to achieve bone and soft tissue healing^3^. Thus, the rate of complications associated with open tibial fractures is high; infection, non-union and limb loss are the major causes of morbidity^4^.

The management of these fractures requires a multidisciplinary approach in order to achieve quick healing and early ambulation for the patient. Various classification systems have been proposed in literature, in an effort to grade the extent of the initial injury, and to offer useful prognostic clues to aid in deciding on the optimal management^4^-^9^. The most widely used is the Gustilo-Anderson classification, which describes three groups of increasing severity based on the size of the open wound, the degree of contamination and the extent of the soft-tissue injury^4^.

Open fractures also result in high rates of infective complications, due to communication with the external environment. As such, antibiotic prophylaxis is usually administered before, during and after intraoperative surgical fixation. Although nosocomial organisms are usually implicated in deep surgical site infection^10^, no study has yet evaluated the organisms grown in all grades of infected open tibial fractures.

One of the aims of this retrospective study is to review the risk factors causing both infective and bony complications in open tibial fractures. An understanding of these risk factors could assist in the formulation of protocols to reduce the rate of complications. Furthermore, this study also aims to collect data on the nature of organisms that cause infective complications, in order to recommend targeted antibiotic prophylactic regimes.

## Materials and Methods

Data was collected over a five-year period from 2006 to 2011. All patients who sustained an open tibial fracture and were treated operatively in a single Level 1 trauma centre were analysed. Exclusion criteria included fractures requiring amputations as forms of definitive management, as well as patients who were lost to follow-up before radiographic bone union was achieved.

The cohort of patients was initially obtained from surgical audit data. A retrospective analysis of online medical records was conducted and epidemiological and clinical data were collected. Parameters gathered included age, sex, the presence of diabetes mellitus, location of fracture, Gustilo classification, and time taken from injury to fixation, infective complications, bony complications, and postoperative tissue cultures in infective complications.

The location of fracture was based on whether it was in the proximal, middle or distal third of the tibial shaft. Infective complications were defined as osteomyelitis, implant infection or soft tissue infection. Bony complications were defined as mal-union, non-union, delayed union or a failure of the implant.

Statistical analysis was conducted using STATA 13 (StataCorp, College Station, TX). Numerical variables were presented as mean ± standard deviation with the student’s t-test, while categorical variables were presented as numbers and their corresponding percentage. The Chi square test or Fisher's exact test were used as appropriate. Variables of p value < 0.2 from the univariate analysis were then selected to be used in the multivariate logistic analysis. A two tailed significance level of 0.05 was used for all the tests.

## Results

One hundred and eighty-five patients were treated at our institution over a five- year period from 2006 to 2011, but 12 were lost to follow-up before radiographic bone union was achieved. As a result, 173 patients were included in this study. Of these, six had sustained bilateral open tibial fractures, with 167 sustaining unilateral fractures. The mean age of the 173 patients was 38.4 years (18-92, standard deviation 14.5). There were 153 males (88%) and 20 females (12%).

Table I illustrates the risk factors affecting post-operative complications in open tibial fractures. Seventeen patients (9.82%) had diabetes mellitus (DM). Twenty-four patients (14.1%) had sustained a fracture located at the proximal third of the tibia, 80 (47.1%) at the middle third, and 66 (38.8%) at the distal third. In terms of Gustilo classification, 31 patients (17.7%) sustained a Gustilo type I fracture, 59 patients (34.0%) a Gustilo II fracture, and 83 (48.3%) a Gustilo IIIa, IIIb or IIIc fracture.

**Table I:** Risk factors for post-operative complications

Variables	Number	Percentage
Diabetes Mellitus		
Yes	17	9.8%
No	156	90.2%
Location of Fracture		
Proximal 1/3	24	14.1%
Middle 1/3	80	47.1%
Distal 1/3	66	38.8%
Gustilo Classification		
Grade I	31	17.7%
Grade II	59	34.0%
Grade IIIa	33	19.2%
Grade IIIb	41	24.1%
Grade IIIc	9	5.0%

In terms of the numbers of post-operative complications, 31 patients (17.9%) obtained a bony complication, while 37 patients (21.4%) sustained an infective complication (Table II). Of these 37 patients, tissue or bone cultures in 40.5% grew Methicillin-resistant staphylococcus aureus (MRSA). Tissue or bone cultures in another 16.2% grew organisms from the Enterobacter genus, while 8.1% grew Serratia marcescens, (Table III).

**Table II:** Post-operative complications of open tibial fracture fixation

Complications	Number	Percentage
Bony	31	17.9%
Infective	37	21.4%

**Table III:** Organisms grown in infective complications

Organism	Number	Percentage
Methicillin-resistant staphylococcus aureus (MRSA)	15	40.5%
Enterobacter	6	16.2%
Serratia marcescens	3	8.1%
Methicillin-sensitive staphylococcus aureus (MSSA)	3	8.1%
Others (Corynebacterium, Coliforms, Enterococcus, Pseudomonas aeruginosa, Citrobacter)	10	27.0%

A multivariate analysis of these results were performed, with the results shown in Table IV. The odds of a patient with a Gustilo type III (a, b or c) fracture developing an infective complication was 3.72 times that of a similar patient with a Gustilo type I or II fracture, after adjusting for the fracture location, time to fixation, age and diabetes (p=0.007). Similarly, the odds of a Gustilo type III fracture developing a bony complication was 4.18 times greater than a type I or II fracture (p=0.015).

**Table IV:** Multivariate analysis

Variables	Infective complications		Bony complications	
	Odds (% CI)	p Value	Odds (% CI)	p Value
Gustilo Classification				
I/II	1.00		1.00	
III (a/b/c)	3.72 (1.44 - 9.6)	0.007	4.18 (1.33 - 13.17)	0.015
Fracture Location				
Proximal 1/3	1.35 (0.41 - 4.47)	0.623		
Middle 1/3	1.00			
Distal 1/3	0.37 (0.13 - 1.1)	0.075		
Time to fixation				
Within 24 hours		1.00		
After 24 hours		0.15 (0.01 – 1.15)	0.063	

The odds of developing an infective complication with fractures of the distal third of the tibia were found to be 63% less than in fractures of the middle third of the tibia, although this was not statistically significant (p=0.075). Similarly, there was no statistical difference in infective complications of fractures of the proximal and middle third of the tibia (p=0.623).

Furthermore, there was no significant difference in the rate of bony complications in patients who had fixation performed for their open tibial fracture within 24 hours of injury, as compared to those with fixation performed after 24 hours (p=0.063).

## Discussion

The Gustilo-Anderson classification has been commonly used in guiding treatment and predicting outcomes of open fractures. Despite some questions about its limited interobserver agreement, it is still the most useful tool to evaluate open fractures ^11^. This study has similarly found a strong correlation between the Gustilo classification of injury of open tibial fractures and the development of complications, which corroborates with current literature. This further highlights the need for increased vigilance, monitoring and regularity of follow-up for patients with a high grade Gustilo injury post-surgical fixation.

The time to fixation after an open tibial fracture has been the subject of continued debate in literature. Choudry *et al* noted that in patients with Gustilo IIIb fractures, early fixation (< 1 week) was associated with a non-union rate of 42%. This was in contrast to a rate of 74% in patients who had surgery later than 1 week from presentation ^12^. Gopal and Tropet independently found that the rate of complications was lower in patients who had soft tissue reconstruction done within 72 hours^13^-^14^. They concluded that injuries with less severe soft tissue trauma are more amenable to earlier soft tissue coverage operations, reducing the risk of osteomyelitis.

However, Franken *et al* demonstrated that there was no significant difference in the rate of bony complications between groups with early (<72 hours) and delayed fixation^15^. Karanas *et al* went on to demonstrate that soft tissue coverage could be performed safely and effectively in the delayed period (>72 hours) with a complication rate of only 7.1%^16^. This study also showed that the rate of infective and bony complications was not significantly affected by the time taken from injury to fixation. Alas, while it is accepted that good results can be achieved even with a delay between injury to fixation, it has to be noted that these results were achieved with optimal wound care and meticulous treatment planning.

In this study, the location of the fracture and time to surgery did not have a significant effect on the rate of complications, but trended towards it. This could be due to our study’s sample size and larger studies are warranted to confirm this relationship.

With regards to the organisms implicated in infective complications, this study found that most of the organisms grown were nosocomial organisms. As such, a single prophylactic antibiotic regimen directed against environmental wound contaminants does not provide cover for the organisms responsible for the majority of postoperative infective complications. Conversely, they may have instead depopulated the fracture site, promoting nosocomial contamination prior to closure. Better wound care and sterile conditions, which prevent the transmission of these organisms at the hospital level, appear to be essential in reducing the rates of infective complications. Various practical measures can be put in place, including screening patients prior to admission, hospital isolation programmes, proper hand hygiene and restricting certain antibiotic usage, particularly fluoroquinolones^17^-^20^.

Traditionally, empirical antibiotic prophylaxis has involved the use of first-generation cephalosporins such as cefazolin (1-2g, 6-8 hourly) for Gram-positive coverage in Gustilo-Anderson type I fractures. An aminoglycoside such as gentamycin (120mg, 12 hourly) is added in higher grade injuries for Gram-negative coverage. Additionally, metronidazole (500mg, 12 hourly) or penicillin (1.2g, 6 hourly) can be added for coverage against anaerobes.

In March 2013, The Antimicrobial Stewardship Group for The Royal Devon and Exeter National Health Service Foundation Trust (UK) recommended guidelines advocating the use of a single regimen of co-amoxiclav (amoxicillin and clavulanic acid) before the first debridement of open fractures of the lower limb. The Surgical Infection Society’s guidelines recommend the use of a short course of firstgeneration cephalosporins^21^. However, our study illustrates the additional need for prophylaxis to be directed at nosocomial organisms. This paper advocates a prophylactic antimicrobial strategy of using a second regimen of prophylaxis against nosocomial organisms, including but not limited to the use of vancomycin. This can be used in addition to the traditional antibiotic prophylaxis for Grampositive coverage, such as with cefazolin.

## Conclusion

The higher the Gustilo grade of an open tibial fracture, the higher the risk of post-operative complications. The location of the fracture as well as time taken from surgery to fixation did not significantly affect the rates of post-operative complications, but results were trending towards significance. Further studies with a larger sample size are warranted to determine the significance of their effect on complication rates. A high rate of post-operative infective complications is due to nosocomial organisms. As such, antimicrobial prophylaxis against both nosocomial organisms and environmental contaminants should be used in order to minimize the rate of infective complications.
